# Factors Influencing the Application of Connected Health in Remote Areas, Taiwan: A Qualitative Pilot Study

**DOI:** 10.3390/ijerph17041282

**Published:** 2020-02-17

**Authors:** Sonia Chien-I. Chen, Chenglian Liu

**Affiliations:** 1 School of Economics and Finance, Huaqiao University, Quanzhou 362021, China; drsoniachen@mail.com; 2 School of Computing, Neusoft Institute of Guangdong, Foshan 528225, China

**Keywords:** connected healthcare, remote areas, business sustainability, population ageing

## Abstract

This pilot study investigated factors influencing the application of connected health (CH) in Taiwanese remote areas. These factors cover issues of cost, infrastructure, technology, business sustainability, business model, collaboration, and communication. It aimed to explore the significance and to assess the feasibility of researching CH in Taiwan. A qualitative exploratory study was conducted by interviewing relevant stakeholders (*n* = 18). The majority were healthcare providers as most of them are the CH end users. Their feedback was essential in reflecting the effectiveness of CH products and services. Therefore, understanding their views is significant in the design of a successful and user-friendly interactive system. A theoretical framework on the introduction of innovations in healthcare was employed to guide data collection and thematic analysis. Additionally, stakeholders proposed strategies for enhancing the implementation of CH in remote areas. This pilot study also contributed to identifying future directions and information for conducting the multi-stage interviews for collecting the data more effectively. Although the results reveal that the study of CH is meaningful, there is an issue of business sustainability which is obscured by some barriers that need to be addressed. These barriers will be further investigated in the first-stage interview and second-stage interview in future research. The research findings also suggest that strategies and sustainability for CH implementation should be included from the planning phase to benefit all the stakeholders in the CH ecosystem.

## 1. Introduction

### 1.1. The Needs of an Ageing Society and Remote Residents

There is a controversy over the concern of rural health disparities in the practice of a democratic society. Some believe that health disparities should not exist in a democratic society due to equality consideration, while others think the power of influence will determine the focus of health facilities dispense. Undoubtedly, more rural areas do present adverse health conditions than suburban areas [1]. To tackle rural health disparities and ageing society challenges, connected health (CH) is proposed as a promising solution worldwide. Where CH is practiced, it usually fulfills the pre-conditions of advanced information technology (IT), integrated healthcare, significant geographic isolation and population of ageing or with chronic conditions.

Taiwanese CH, as a case study, is significant and valuable as it demonstrates typical features which may impact and interest the whole of society. Taiwan has become one of the fastest ageing populations in the world due to advancements in medical technology, changes in family structure, and the low birth rate [2]. Over the past century, there has been a dramatic increase in life expectancy accompanied by a growth in chronic conditions (such as hypertension, diabetes, coronary diseases, and obesity) [3]. Geographically, Taiwan’s remote areas cover mountain areas, isolated islands, and remote townships [4]. In these areas, medical and care services are allowed to be practiced remotely according to the law and regulation of the state. There are 48 remote townships that account for 44% of the area in Taiwan, while their residents are only 0.36 million people which accounts for 1.6% of the total population [5,6,7]. These features make Taiwan a typical case to study CH’s challenges and strategies in remote areas accompanied by ageing populations. 

Much health-related research is conducted through quantitative method due to its evidence-based nature. Quantitative research is designed for the natural sciences and covers surveys, laboratory experiments, econometrics, statistical analysis and others. On the other hand, qualitative research methods are developed in social sciences for the study of social and cultural phenomena [8]. It features on seeing the world from the participants’ lenses to discover insights that will contribute to the development of empirical knowledge. Although this study was conducted in a qualitative manner, which differs from a qualitative research that can offer a statistics variation, it may contribute knowledge by offering insights and implications that may inspire areas that encounter similar challenges [8,9]. Based on findings of this study, a further discovery of the root causes of the issues in CH can be identified. Their connection with business model innovation can be developed as an innovation driver to sustain CH’s practice.

Studies of Taiwanese universal health insurance (UHI) are of interest for researchers due to the comprehensive coverage of care for residents and the reduction of health disparity in the society [10]. Particularly, the Taiwanese society has distinct universal health insurance that covers all citizens both in remote and urban areas. However, significant benefits for the insured elderly among remote residents need to be explored and addressed. In the past, challenges in accessing health care associated with transportation, social isolation, poverty, and a lack of health care providers, especially medical specialists confronted the rural residents to facilitate the benefits of UHI, CH is expecting to make contributions to equitable right to access healthcare. What is disputed in the democratic society is the right of equality and the contributions of its participants [11,12]. It is seen that citizens pay for the same insurance fees, while not everyone enjoys the same health benefits. Although the implementation of health facilities in remote areas may be costly, it is relevant to concern oneself with the needs of remote residents in a democratic society [13]. Therefore, the authors aimed to identify key challenges and possible strategies in CH to ensure the quality of care and equality. It is expected that the finding of this research can benefit an ageing society and remote residents. The objective of this study was to discover CH’s influencing factors so as to develop strategies of removing barriers and improving access to health services for remote residents.

### 1.2. The Selection of Research Target

Several essential features are identified to conduct a meaningful connected health study based on literature review [14,15,16]. These are demographics of population ageing; comprehensive CH ecosystem, Information Communication Technology (ICT) infrastructure; and the region or area with significant geographic features. Taiwan has been selected as a critical case study as it meets the preconditions and essential requirements for the successfully implementation of CH. Taiwan has its importance uniqueness, and revelatory in researching CH due to the following characteristics: Taiwan has become one of the fastest ageing population areas in the world due to the advancement of medical technology, changing family concepts, and a low birth rate as well as its significant geographic features [17,18,19,20,21]. 

**Figure 1:**
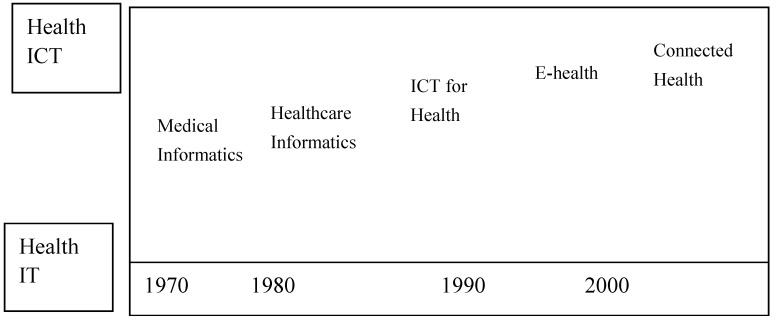
The evolution of names from ‘medical informatics’ to ‘connected health’ (Rossi Mori et al., 2007).

Although in Taiwan, different terms, such as remote health, Telecare or Telehealth, in general, are more frequently used rather than the term “connected health” (CH) for describing similar models by healthcare professionals, it is still in the same domain for discussion and comparison (Figure 1) [22,23,24,25,26,27]. Currently, researchers tend to use Connected Health as a term that applies to the linked systems and people in the application of ICT in healthcare in general [14,28,29].

The literature indicates that CH includes the whole Telemedicine (TM) family: Telemedicine (TM), Telehealth (TH), and Telecare (TC) [14,28,29]. Although Taiwanese Telecare pilot schemes have proven the feasibility of TC, there were not many remarkable results of cost-saving from TH and TM. Moreover, the cases of successful business models (BMs) still remain open issues. The results of Taiwanese pilot schemes were varied. Among these issues of CH, payment models are one of the most important issues as they play an important role in keeping CH businesses sustainable. The CH in Taiwan, like many other areas, have an issue of being sustainable after government funding has finished. Designing comprehensive BMs becomes the key to success.

According to Pare et al. [30,31], the results of a systematic review of home telemonitoring, after surveying 65 programs across North America, Europe, and Asia, show the feasibility of telemonitoring healthcare [32]. However, the results for Taiwanese telemonitoring programs from 2007–2009 have shown that Taiwanese programs are competitive in terms of patient acceptance and satisfaction; a decrease in hospital admissions and a decrease in emergency room visits (shown in Table 1 and Table 2). 

The reasons, such as “preventing ill health”, “maximizing the potential of technology”, and “supporting workforce” were those which drove the Telehealth (TH) pilot project in Taiwan [33]. It was initially executed in 2008 and within one year, three different models had a noticeable performance:

1. In the home care model: the hospital readmission rate had a reduction of around 61%; the hospital visit rate had a decrease of over 1%;
2. In community care models, the medication non-adherence rate had a decline of over 76%;
3. In the residential-care model, the readmission rate to the hospital was reduced by over 25%, nosocomial infection decreased by over 38%, and the drug duplication rate declined to 53% [34].

The concept of this TC monitoring is to allow the long-term management of chronic illnesses as well as providing immediate information, to give an early warning of possible diseases—both of which meet the goal of preventive healthcare. 

People in Taiwan tend to share the same feeling of being “Aging-in-Place”, which offers remote health a strong reason to develop [35,36,37,38]. Pressure on financially constrained healthcare systems is exacerbated by the stress of heavy workloads among healthcare staff. Irrespective of the national effort involved, the wish of the Taiwanese democracy is to maintain and improve its universal healthcare system, but hard work alone cannot maintain it anymore. Therefore, what are the options when putting in more human effort is no longer an option? 

**Figure 2:**
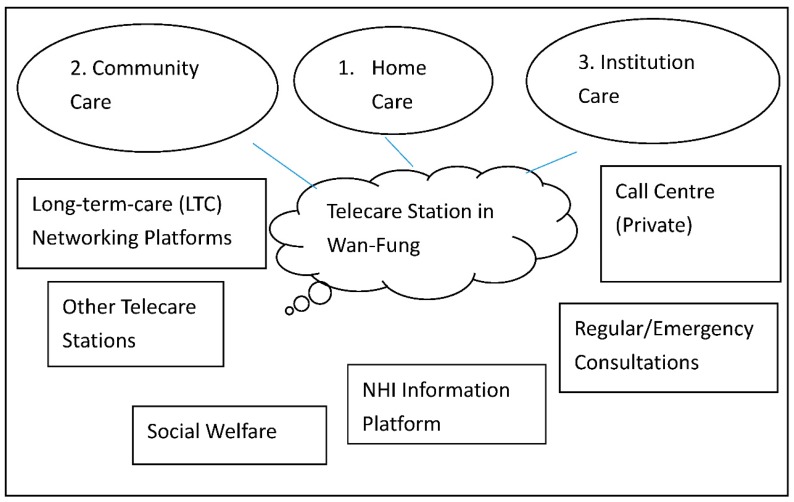
Telecare (TC) in Taiwan (source: Department of Health, Taiwan, 2009).

In the TC pilot project, an electronic care records exchange system and an authorized environment were constructed under three service models (home care, community care, and institutional care) in order to offer more integrated care services (shown in Figure 2). These three models are classified and featured according to where the care is facilitated. The home care is mainly supported full time by the family members of the care receivers while the community model is supported by community facilitators in the daytime and family members after their working hours. As for the institutional model, it is commonly for those who cannot be supported by their family and community members. They will be cared by the institution required in a fulltime base, such as a care home. In general, these three models work independently. However, they will seek help from each other once they are in need. The community care service model aims to establish a community model by connecting various community medical care service resources. The home service model seeks to introduce more than 150 home subjects and connect more than five home care service resources. The institutional care service model cooperates with hospitals and nursing homes to construct two nursing home models. In order to support and monitor quality customer service, 24-hour call centers are established. Long-term care (LTC) networking platforms are built to offer integrated, seamless care by connecting subjects’ information from different care service providers with a referral system to increase resource connecting efficiency. Many educational and promotional events are held to spread the word about these three care service models in order to encourage their potential duplication and expansion.

While some researchers argue that the incorporation of new technologies may not contribute to patient satisfaction, a successful Taiwanese pilot project has proven that this application is beneficial for building up trust and mutual understanding between patients and hospitals and also for reducing criticisms of hospitals [39,40]. This project has also proven that TC is effective in maximizing the potential of technology in addition to showing several other immeasurable benefits, such as allocated medical resources and preventive health [14].

Although this government-sponsored platform can enable tele-monitoring and patients’ self-management to allow remote residents living independently, some issues remained to be addressed. Compared to the institution care model, community care and home care models are more culturally accepted among these three models. As the concept of living with parents and taking care of parents at home are the mainstream in Taiwanese society. To promote the facilities and benefits of institutional care may be an opportunity of action. With the shift of family concept, institutional care may become more and more population in the future. For the other two models, the cost issue may be the most relevant factor influencing people using CH facilities. Most of the users are happy about the services, but only a few people are willing to pay for them. It is suggested that developing a comprehensive business model will be critical for sustaining the CH system of home and community model. What they have in common is that concerns of system integration, information security, and multidisciplinary collaboration need to be resolved among these three models. These challenges will be further communicated in the Result and Discussion Section. The findings of Taiwanese experience imply that users, especially older adults are seeking the care environment that can allow them to live with dignity and comfort. Therefore, it is relevant to understand the key factors of CH’s service models for the benefits of promoting and developing the CH industry. 

### 1.3. Conceptual Framework

**Figure 3:**
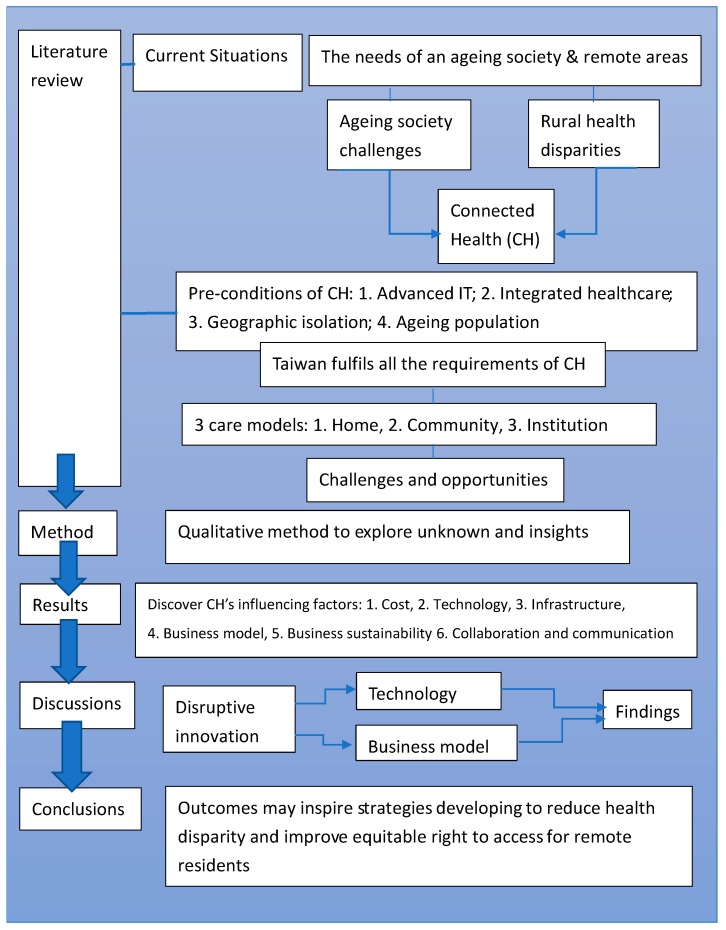
A conceptual framework of this study.

A conceptual framework was developed according to the literature review and will be further developed accordingly after the data analysis, as shown in Figure 3. The initial issues of CH have been identified in the literature review and further CH challenges have been assessed through data collection and analysis. 

According to the three care models mentioned above, they reveal that the institutional care model has potential to be expanded; a sustainable BM needs to be developed to resolve issues of system integration, information security, and multidisciplinary collaboration. Specifically, these issues can be concluded as costs, infrastructure, technology, business model, communication and collaboration, and business sustainability. A further study can be designed based on the characteristics of the factors related to CH in the results section, so that effective investigations of next stage can be developed and followed up. 

In the discussion, the disruptive innovation theory and relevant literature are employed to communicate with the findings. Key factors were identified and classified into two groups: generic to health systems and specific to CH inclusion. Generally, it seeks to discover how current health infrastructure can support the implementation of CH. Specifically, it aims to investigate how technology can mediate between geographic isolation and accessibility and how a business model can interact with cost and purchase capability/affordability. Business model and business sustainability are also crucial factors to be discussed, as both direct cost and indirect cost are relevant factors influencing remote residents’ affordability. Therefore, it is relevant to understand the key factors influencing the application of Connected Health in remote areas in Taiwan with a qualitative pilot study for the benefits of promoting and developing the CH industry.

This pilot study aimed to identify future directions and information for conducting the multi-stage interviews for collecting the data more effectively. According to the finding of this study, a comprehensive research for investigating CH’s sustainability and potential strategies that may contribute to academia, industry and government can be designed in the next stage.

Research questions:

(1) What are the factors influencing the implementation of connected health in Taiwanese remote areas as perceived by stakeholders?
(2) Which factors are perceived by stakeholders as specifically important to the adoption, implementation, and continuation of connected health in remote areas?
(3) What are the perceived facilitators and barriers for users in remote areas to access healthcare service through technology?

This research topic is valuable and valid as it influences a human being’s life to such a significant degree. It is particularly important when we are facing global economic and social challenges. It could possibly transfer threats into opportunities. It potentially can generate knowledge that will make contributions to improving efficiency and effectiveness in the healthcare sector. 

## 2. Methods

### 2.1. Participant Selection

Stakeholders and experts were selected for the interviews according to the literature review and snowball sampling [41]. These participants were interviewed on a voluntary basis and gave verbal consent according to ethical guidelines. This study was approved by Ulster University’s institutional review board. The reference no. is RG3 RMcAdam2. The study’s participants included healthcare professionals, industry players, academic researchers, and government agents. The researchers identify participants’ backgrounds, competencies, gender, their geographic locations, etc., as they may relate to the outcomes of this research. Our semi-structured interview guide comprised the following:

- Working sectors;
- Background information;
- Gender;
- Levels of competency.

### 2.2. Justification of Method Used

The reason why the qualitative method was employed in this study is justified here through the comparison of quantitative and qualitative research, as shown in Table 3. According to Bryman, contrasting aspects of quantitative and qualitative research is presented to demonstrate the main features of these methods [41]. The former focuses on numbers and a statistics variation to test theory from the point of researcher while the latter emphasizes the meaning of words or phrases to emerge theory from the point of view of participants. This study aimed to discover and explore factors influencing the application of CH in remote areas, it closes to the nature of obtaining contextual understanding of qualitative research from rich and deep data. The most important one is the feature of seeing the world from the participants’ lenses to discover insights that will contribute to the development of empirical knowledge. As this study seeks to obtain the inner experience of participants, to determine how meanings are formed through and in culture rather than test variables, which meet the features of the qualitative approach. Qualitative research is fluid, evolving and dynamic in nature; on the contrary, a quantitative approach is more rigid and structured in format. Statistics might be interesting; however, exploring the infinite possibilities of targeted participants may be more attractive to qualitative researchers [42]. Therefore, qualitative research is more suitable to this study. 

### 2.3. Data Collection

In this pilot study, an exploratory approach was conducted involving semi-structured in-depth interviews with 18 participants from remote allied case studies and their stakeholders. “Four administrative areas of applied CH based on geographic and rural location” was the main criteria for selecting participants. “Those who have experiences in CH” was the second selection criteria. These sampling criteria were chosen in order to include the inputs of major and significant participants with various levels of rural experience and management that can be covered. Consequently, health institutions covering from northern, central, southern, and eastern Taiwan were covered in this study. 

Following the informed consent, researchers conducted face-to-face interviews with seventeen participants from 30 min to 2 h. Being aware of the significance of validity and rigor of qualitative research, several research strategies were conducted to ensure the quality of data collection. These protocols cover the use of the Agile method to allow interviewees’ opinions to be validated and exchanged through a semi-structured interview manner. Any differences between the interviews were discussed until a consensus was reached. Audio-taping interviews and transcribed verbatim by an independent typist were subsequently validated by the researcher. Meaningful quotations were adapted to represent important themes. To address participants’ confidentiality, data were processed through de-identified considerations. 

#### 2.3.1. Pilot Interviews

There were 18 interviewees to be chosen in the pilot interviews (as shown in Table 4). They were chosen according to CH literature and snowball sampling, which were classified based on working sectors, background information, gender, and levels of competency. The age indicator is not included as this study considered that competency is more relevant than age and can be covered by competency. Apart from that, most of participants were between the ages of 40 and 50; therefore, it does not show significant difference in this study. Although the sample size is small, it covered representative participants in the microenvironment. In the pilot study, the variety of participants are more important than the numbers. A bigger population size and a more uniform distribution will be made in the next stage of research. The majority were healthcare facilitators as they were inevitable in the CH ecosystem. Their feedback was significant in reflecting the effectiveness of CH products and services. Therefore, understanding their views was significant in the successful and user-friendly interactive system design. This pilot interview focused on health professionals rather than patients and their families due to ethical concerns.

#### 2.3.2. Data Analysis Methods

Data are concurrently collected and analyzed until theoretical saturation is reached as suggested by Yin [41]. In this research, a thematic and a systematic approach to qualitative analysis was performed collaboratively. The thematic analysis was organized via deep familiarization with the data collected leading to the development of major themes. The systematic analysis was employed as quasi-statistical (content) analysis to ensure the validity of the analysis. These themes were further analyzed to identify sub-themes and were structured to provide a comprehensive account. 

The author adopted a data triangulation testing method to ensure that the analysis of the results was valid. Data triangulation examined the consistency of different data sources with the same method. They can be at different points in time and space; allowing for the comparison of people with different viewpoints. This included interviews taking place at different points in time and with four different entities (such as government, academia, industry, and healthcare providers). The theory triangulation included a content analysis, a template analysis, and an editing analysis (thematic analysis), which led to the use of multiple theoretical perspectives to analyze and interpret the same phenomenon in this research. 

In this study, data were categorized using a thematic analysis. Codes were derived from the data through several steps, including data cleaning, data summarizing, data analysis, and data mining. The categorization of data consisted of continually revisiting and reviewing until the themes and categories used to summarize and describe the findings were verified and accurately reflected the data. A qualitative data management system, the Nvivo 12 software (produced by QSR International, Melbourne, Australia), was employed to manage the data throughout the process. Firstly, data were cleaned through the integration process to merge different terms which have the same meaning. For example, connected health could be called remote health, telehealth, and telecare in the interviews. Terms were merged according to the actual meaning of interviewees. Secondly, data were summarized, clustered, and categorized based on the meaning of interviewees. Thirdly, data were analyzed and extracted according to the insightful meaning from interviewees as it is the stage of the data mining process. For example, some issues were raised by interviewees but the meaning behind the issues and the root cause of the issues need to be analyzed.

## 3. Results

### 3.1. Pilot Interview Results

A sample of 18 respondents was interviewed. These covered key influencers, including government, academics, health professionals, and relevant business professionals. They were selected through voluntary participation and a significant sampling method across various health system levels. The purpose of the pilot interviews was to identify factors influencing application of CH in remote areas. This pilot study also has the advantages of identifying potential directions and information for conducting the multi-stage interviews for collecting the data more effectively. Although the results reveal that the study of CH is meaningful, there is an issue of business sustainability, which is obscured by some barriers that need to be addressed. These barriers will be further investigated in the first-stage interview.

**Figure 4:**
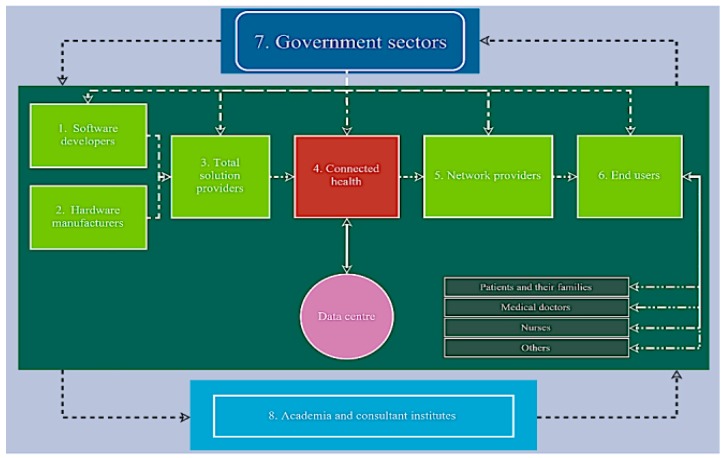
CH players and stakeholders’ relationship diagram [43].

Figure 4 and Table 5 illustrate the relationships and collaboration between CH players and stakeholders in its business ecosystem [43]. Interviewees are selected and grouped into eight categories based on this business ecosystem. What Figure 4 presents is the relationship diagram among CH players and its stakeholders. It is seen that a CH ecosystem should cover Government sectors (No. 7), Industry players (No. 1–6) and Academic and consultant institutes (No. 8). What is noticeable is that Connected Health service provider (No 4) is in the central position that interacts with government and industry. It also communicates with data centre for data storage and retrieval. There is a variety of End users (No. 6), they can be patients and their family, health professionals and others according to the circumstance that occurred. As for the Academia and consultant institutes (No. 8), they play a role of providing fundamental knowledge for CH players to act upon. Although it seems to be relatively passive and invisible, it is evitable in the system. Due to the limitation of the current regulation, CH can only be practiced in hospitals or care institutions in Taiwan. Although they are difficult to define as academia or industry, they are important roles in this field. By collecting different perspectives in this research, it aims to inspire more creative approaches to exploit the innovative advantages accruing from healthcare innovations.

Table 6 presents the results of the CH issues that are scored by the interviewees. Interviewees were numbered (101, 201, …) based on the eight categories from Table 5. If the issue is considered “important”, it gets one point. If the interviewees think the issue “may be important”, it gets 1 point, if they are “not sure” about it, it gets 0 points. If the issue is considered as “not important,” it gets −1 point. The table sums up the results to see the importance of CH issues identified in the stage one data collection (shown in Table 6). This table measures the importance of each category according to participants’ perspective. The description of each category is presented in Section 3.2.1, Section 3.2.2, Section 3.2.3, Section 3.2.4, Section 3.2.5 and Section 3.2.6.

### 3.2. Characteristics of the Factors Related to CH

What influences the implementation of connected health in Taiwanese remote areas varies in regard to the nature of stakeholders based on Table 6. Factors are explored with interviewees from government, industry, academia, and health institutions. They identified factors relating to CH in Taiwanese remote areas as determinants of successful adoption, implementation, and continuation. Factors are scored by importance in Table 6 according to respondents’ viewpoint. They reported that the engagement of remote residents, reachable health service infrastructure, accessible methods of communication, and the availability of human resources and equipment were all important characteristics of CH projects in remote areas. According to Table 6, the factor of technology and business model obtain the highest score, while the factor of collaboration and communication comes next. The factor of infrastructure seems to be less important for participants. Health facilitators of remote residents highlighted factors relating to inclusive costs, infrastructure, technology, business model, communication and collaboration, and business sustainability. Their characteristics are described below.

#### 3.2.1. Cost

Initially, the Taiwanese government sponsored several pilot schemes to assess the feasibility of CH in remote areas. It can be seen that the efficiency of care can be delivered through these services. However, after the sponsorship ceased, residents had little interest in using CH services as they have a limited purchase capability. Alternative solutions should be provided to sustain the business.

*“Remote residents are usually voluntary groups; they have low purchase capability … They often rely on physicians to visit or public resources to manage their health.”*

(General physician, male)

As far as cost is concerned, some health institutions request that their suppliers offer a free trial for a period of time to initiate these businesses. The industrial players need to take the risk first before getting the payment from customers.

*“Hospitals request a free trial from us to motivate their end users, … once they are not happy about the products, we cannot make any money.”*

(Business manager, male)

In the CH industry, health professionals have authority over customers. If the products are recommended by them, customers are more likely to adapt them. Therefore, companies prefer to partner with hospitals. However, hospitals usually do not want to take the business risk for them, so they will ask for a free trial prior to payment to reduce the cost burden for consumers.

*“Health professionals have a strong authority over end users, so we need to work with them, even though we might need to sacrifice some benefits.”*

(CH tech adviser, male)

#### 3.2.2. Infrastructure

Infrastructure refers to the essential requirements for constructing CH platforms. As the literature suggests, CH requires collaboration from the government, academia, and industry to build a comprehensive ecosystem. Although cost issues are important, without essential infrastructure, CH services cannot be provided, especially as they are closely related to national infrastructure. Reducing the cost of traveling to remote areas is one of the most proposed features of CH but many remote areas currently lack the ICT infrastructure necessary to implement CH services. Low population density makes it less cost-effective to build up infrastructure, such that profit-oriented companies are likely not to be eager to adopt it. In this case, government intervention is necessary.

*“Network companies think building up infrastructure in remote areas is not cost-effective, as the scale of economy is too small, they are not willing to make efforts on it … Without the government’s intervention, CH infrastructure is difficult to be accomplished.”*

(General Physician, male)

*“Although infrastructure is essential, there is nothing we can do about it… maybe the government has the power and authority to do it,… what we can do is to design a product that relies on current resources as much as possible.”*

(Manager, male)

#### 3.2.3. Technology

Technology issues refer to the technical challenges based on the current infrastructure. It not only enables CH in practice but also ensures accurate and reliable data that can be converted into useful information. The focus has been on vital sign parameter measurement management and audio/video consultations technology. In remote areas, internet infrastructure is very expensive. According to research, it usually requires 5 to 10 years to earn back initial expenditure, such that it is often not considered cost-effective for ICT companies to establish infrastructure. In addition, many people still have fear and uncertainty in relation to new technological interventions. Therefore, the concern needs to be with user-friendly customer-focused infrastructure.

*“Building infrastructure is very expensive. We aim to offer an all-inclusive service through our connected device, in this case, users do not need to rely on the government’s infrastructure but current resources …”*

(Manager, male)

*“We use the technology of smart house to monitor residents’ health and connect to health institutions.”*

(Social worker, female)

Apart from this, associated standards such as data exchange security, safety, and privacy, are essential to take into account. Controversy has continued over the feasibility and customer satisfaction within CH practices as it has been argued that there is both a lack of evidence and a lack of confidence in the successful implementation of CH. The issues of resistance to change and a slow clinical acceptance of CH remain.

*“I have been a general physician for over a decade, and I only believe personal contract, any what is called technology in health, is only a buzz word for me!”*

(General physician, male)

What matters to health professionals is whether technology can achieve their mission to improve the care experience, enhance outcomes, and reduce costs rather than the means that technology is addressed. Health professionals want technologies that can be deeply embedded into their workflow and be situated in one location rather than increase their workload. This implies that technology not only plays the role of solution provider or enabler but, most important, that it should also integrate all players in pursuit of customer value and a user-friendly interface and design.

*“Our clinic is open to all the solutions that are beneficial to our patients. We are happy to accept all the new technology as long as it works.”*

(Director/General physician, male)

#### 3.2.4. Collaboration and Communication

As CH involves roles in many different sectors and departments, the versions of CH and the interests of stakeholders may be diverse. Therefore, the authority and the executor may have different opinions on the same project. Support from the top is important; however, how to integrate resources and to build up cross-department collaboration remains a challenge to be overcome.

*“I only know our part of the business and take orders from my supervisor. I have no clue about the business from other sectors …”*

(Tech advisor, male)

*“CH can do more things than we have known today, especially the holistic healthcare management … that is why our government wants to integrate the department of health with social welfare to enable more integration and cooperation.”*

(General physician, male)

Quality problems are caused by poor integration. It is essential, therefore, to carefully consider issues of integration when planning or implementing a healthcare service that seeks improvements both to quality and cost-effectiveness. The importance of communication-related challenges by pointing out that participants in CH have different interests and concerns makes it more difficult to speak in a common language or to share common ground.

*“Our institution aims to offer a more integrated service to help people to find suitable facilitators to solve their problems as we understand many health provisions are isolated from departments …”*

(CEO, male)

*“… an ideal project-based management that can integrate resources is essential to deliver comprehensive services to the public.”*

(Vice-project manager, female)

#### 3.2.5. Business Sustainability

CH businesses encounter challenges related to their sustainability after government funding ceases. Many government-led pilot schemes have pointed to the feasibility of CH and the satisfaction of its clients, but some schemes have proved unsustainable after governmental funding has ceased. Although people have high expectations of CH, sustainability issues remain a major challenge—in the pilot scheme period, the government-sponsored CH expenditure. However, after sponsorship ceases, CH businesses encounter sustainability issues. Some businesses close down while others only barely survive. This may be because of the homogeneity of CH’s business models; although every company has its unique business model, BMs tend to be a similar “pay for service” structure in the case of CH.

*“We have contacted as many CH users as possible, but they are only willing to use our products in the condition of government’s sponsor … They stop using our products once the funding from the government ceases.”*

(Manager, male)

*“This business is not as promising as people seen in the public media, as many of us encounter the issue of business sustainability.”*

(Manager, male)

#### 3.2.6. Business Model

A comprehensive business model not only seeks to solve business sustainability issues but also the other issues mentioned above. Crucial principles and themes of successful implementation of technology-driven healthcare innovation remain to be investigated; agreement needs to be reached and knowledge disseminated. Solutions that promise cost-effectiveness and efficiency may meet the needs of the market. Business modeling of CH care and a social care model may be able to meet these goals.

*“A comprehensive business model under the current health infrastructure may be helpful to keep the connected health business sustained.”*

(Manager, male)

*“I think to make connected health successful is not only about health facilitators but also how to design a comprehensive business model to couple with current stakeholders.”*

(Pharmacist, female)

## 4. Discussions

This study addresses what factors influence the application of connected health in Taiwanese remote areas. The influence of the adoption, implementation, and continuation of connected health in remote areas is informed. As there was a significant overlap in the factors identified based on the responses from these groups, we combined responses of both types of respondents when reporting results. Technology and business models (BM) are perceived by stakeholders as specifically important to the adoption of CH in remote areas. 

Key factors were identified for the introduction of CH at two groups: (1) generic to health systems: infrastructure and collaboration and communication; (2) specific to CH inclusion: costs, technology, business model, business sustainability. These factors highlight the fact that CH in remote areas is achievable with the help of leadership, management, and strategic planning.

The findings suggest that geographic isolation and purchase capability were key restraints for participants. Therefore, how technology and business model mediated between purchase capability and geographic isolation should be highlighted. Health facilitators also indicate that those who seek or accept CH services due to not being able to afford the direct costs and indirect costs (e.g., transport and any accompanying person) may benefit from disruptive technology and business model innovation. These findings are similar to Hwang and Christensen’s perspectives on how disruptive technology can leverage challenges to opportunities for remote residents and why strategies for remote area should be included from the planning phase of a connected health program. [42,43,44]. CH reduce the inability to access healthcare due to poverty increases the risk of poor health. In remote areas, chronic conditions further exacerbate this interaction between poverty and health [45,46]. People with chronic conditions are at greater risk of poverty due to lower employment rates and lower educational attainment [45,46,47,48]. Furthermore, this risk leads to greater inequities in accessing healthcare and having their healthcare needs met, including those relating to chronic conditions, compared to the broader population. Thus, CH is critically needed in remote areas. 

The findings of this study indicate that CH can reduce the indirect costs in remote settings. As at a higher risk of poverty, people with chronic conditions may also incur higher indirect healthcare costs [48]. The need for an accompanying person to enable an ageing population to attend health services was underlined [49,50]. In addition to the transport and other costs for the accompanying person, their opportunity cost of income-earning activities creates a greater risk of household poverty, perpetuating the interaction between poor health and low purchase capability for the person with chronic conditions and their household [51,52,53].

Although those who are close to communities, with the availability of national insurance or a poor certificate, may get reduction of healthcare cost, it is relevant to have an alternative provision of healthcare that can reduce the geographic barriers. These discoveries confirm the needs of a CH study in remote areas. Addressing the factors such as cost, transport, and health workers’ availability through CH are therefore important for providing healthcare to remote residents. Moreover, CH platform can also be used and transferrable for mental health or various age groups [54,55,56]. This study confirms the feasibility of CH in remote areas and seeks to explore deeper its scalability and sustainability in the next stage investigation. 

## 5. Conclusions

This pilot study in Taiwan provided the opportunity to learn what is feasible for CH in remote areas. This study found that factors influencing the adoption, implementation, and continuation of the CH in Taiwanese remote areas were costs, infrastructure, technology, business model, business sustainability, collaboration, and communication. The factors of technology and business model are considered more important than others. These findings echo the theory of disruptive innovation that disruptive technology may leverage healthcare into a more affordable and accessible service. The findings also confirm the needs for a CH study in Taiwanese remote areas and they highlight that strategies development for the remote area should be included from the planning phase of a connected health program. Knowledge of the CH platform can also be used and transferred for mental health or various age groups. This study confirms the feasibility of CH in remote areas and seeks to explore deeper into its scalability and sustainability in the next stage investigation. Information from this research can be used to develop a larger-scale investigation to obtain business vision in future work. 

## 6. Limitations

This study is limited by the sample of participants and ethical concerns; patients were therefore excluded from this study; however, their opinions are reflected by their health facilitators. Some relevant people in CH such as IT security were not included as their influence on the CH system and service is low. Other people such as total solution providers are missed due to the time limitation and the nature of a pilot study and may be covered in the next stage of the interview. After having explored the factors and feasibility of CH in remote areas, another stage of interview is required to get the management’s insights and depth of the research.

## Figures and Tables

**Table 1:** Comparison of telecare results in Taiwan with the combined results of North America and Europe.

Characteristics	Taiwan *n* = 8	North America and Europe *n* = 65
Study period	2007–2009	1990–2006
Duration of each single study	6–18 months	5–12 months
Patient symptom detection	Good	Good
Improved physiological values	Yes	Yes
Patient acceptance and satisfaction	High *	High
Decrease in hospital admissions	Yes	Yes **
Decrease in emergency room visits	Yes	Yes **
Improved medicine consumption	Yes	Unknown
Reduction in medical costs	Yes	Not clear

Source: Lin, 2010. * = in the case of free services, ** = except for patients with diabetes.

**Table 2:** Comparison of connected health (CH) in the US and EU.

CH	US	EU
Stage	Developed, mature.	Initial, pilot.
Service	Remote monitoring, such as remote consultation, video conferencing, and self-management via vital signs parameter measurement, helping patients adhere to medication regimens, and reducing referral wait times.	Self-management, such as with the aim of preventing fall injuries among the elderly. Remote monitoring, such as via alarms, general nursing tasks, physical therapy, social work, nutrition and health consultation, meal delivery, patient transportation, and emergency help provision.
Samples	Up to 18,000.	Up to 6000.
Main segment	Chronic conditions, such as depression, diabetes, heart failure, and Intensive Care Units (ICU).	Diabetes, heart failure.
Public or private	Mainly private.	Mainly public.
Leader	Mainly hospital-led, some are government-led.	Government-led.
Coverage	Nationwide for veterans only, others are regionwide.	Regionwide.
Care model	Institution, home.	Institution, home.
Outcomes	Significant outcomes in quality improvement and cost savings.	Outcomes are varied and limited to small populations.
Business model	Mainly privatized.	Mainly government sponsored.

Source: Summary of literature review by the author.

**Table 3:** Contrasting quantitative and qualitative research.

Quantitative Research	Qualitative Research
Numbers	Words
Point of view of researcher	Point of view of participants
Researcher is distant	Researcher is close
Theory testing	Theory emergent
Static	Process
Structured	Unstructured
Generalisation	Contextual understanding
Hard, reliable data	Rich, deep data
Macro	Micro
Behaviour	Meaning
Artificial setting	Natural setting

Source: Bryman, 2008, p. 393 [41].

**Table 4:** Pilot interviewees’ profile.

	Organization	Gender (M: 56%/F: 46%)	Title/Level of Competency
Industry (4) (22%)	June Sun Digicom	M	1. General Manager
	DigiO2	M	2. Tech Advisor
	Netown Corporation	M	3. Manager
	Far EasTone Telecommunications	M	4. Manager
Government/Semi-government (2) (11%)	Taiwan forces for Medical Travel	M	5. CEO
		F	6. Vice Project Manager
Academia (1) (6%)	Taipei Medical University School of Gerontology Health Management College of Nursing	F	7. Assistant Professor/Attending Physician
Healthcare providers (11) (61%)	Chang Gung Memorial health Village	F	8. Staff
	Antai Medical Care Hospital	M	9. Director/G.P.
		F	10. General Physician (G.P.)
	San-Chung Health Center	F	11. Head Nurse.
	Y.R. Chinese Medicine Clinic	M	12. Doctor
		F	13. Manager
	Tri-service General Hospital	M	14. Medical doctor
	Taiwan University Hospital	M	15. Medical doctor
	Kaohsiung Municipal Hsiaokang Hospital	F	16. Pharmacist
	Zhang Bi Zheng Family Physicians Clinic	M	17. Director/G.P.
	Home Physics	M	18. Therapist

**Table 5:** Interviewees’ categories.

Categories		No.	Name
1	Software developers	101	June Sun Digicom
		102	DigiO2
2	Hardware manufacturers	201	Netown Corporation
3	Total solution providers	301	Not Applicable
4	CH care service providers	401	Chang Gung Memorial health Village
		402	Antai Medical Care Hospital
		403	San-Chung Health Center
		404	Y.R. Chinese Medicine Clinic
		405	Tri-service General Hospital
		406	National Taiwan University Hospital
		407	Kaohsiung Municipal Hsiaokang Hospital
		408	Zhang Bi Zheng Family Physicians Clinic
		409	Home Physics
5	Network providers	501	Far EasTone Telecommunications
6	End users	601	Not Applicable
7	Government sectors	701	Taiwan forces for Medical Travel
8	Academia	801	Taipei Medical University School of Gerontology Health Management College of Nursing

Source: Summarized by the authors.

**Table 6:** Interviewees’ perspectives score in terms of CH issues.

	Cost	Infrastructure	Technology	Business Sustainability	Collaboration and Communication Related	Business Model	Others
101	−1	1	1	1	0	1	Trend of ageing and low birth rate
102	0	1	1	0	0	0	Technology is there to use
201	1	0	1	1	1	1	Mobility and integration of devices
301	0	0	0	0	0	0	It is replaced by 101 and 201 in this study.
401	1	0	1	1	1	1	Human care
402	1	0	1	0	1	1	Government’s incentive matters
403	1	0	0	0	1	1	Government’s policy matters
404	1	0	1	1	1	1	Willing to collaborate once the solution is ready
405	1	1	1	1	1	1	Fellow Government’s policy and projects
406	1	1	1	1	1	1	Holistic health matters
407	1	1	1	1	1	1	CH is essential but an economic scale is requested to sustain this business
408	1	0	1	0	1	1	Happy to accept all the solutions for the benefits of patients and businesses
409	1	0	1	1	1	1	Holistic health matters
501	1	0	1	0	1	1	CH has great potential
601	0	0	0	0	0	0	Their opinions are reflected by health professional.
701	1	1	1	1	0	1	Expand Taiwanese models worldwide
801	0	1	1	0	1	1	Universal design application matters
Total	11	7	14	9	12	14	

Important: 1, maybe or not sure: 0, not important: −1.
